# Metathesis in the generation of low-temperature gas in marine shales

**DOI:** 10.1186/1467-4866-11-1

**Published:** 2010-01-20

**Authors:** Frank D Mango, Daniel M Jarvie

**Affiliations:** 1 Petroleum Habitats, 806 Soboda Ct, Houston, Texas 77079, USA; 2 Worldwide Geochemistry, 218 Higgins Street, Humble, Texas 77338, USA

## Abstract

The recent report of low-temperature catalytic gas from marine shales took on additional significance with the subsequent disclosure of natural gas and low-temperature gas at or near thermodynamic equilibrium in methane, ethane, and propane. It is important because thermal cracking, the presumed source of natural gas, cannot generate these hydrocarbons at equilibrium nor can it bring them to equilibrium over geologic time. The source of equilibrium and the source of natural gas are either the same (generation under equilibrium control) or closely associated. Here we report the catalytic interconversion of hydrocarbons (metathesis) as the source of equilibrium in experiments with Cretaceous Mowry shale at 100°C. Focus was on two metathetic equilibria: methane, ethane, and propane, reported earlier, Q (K = [(C_1_)*(C_3_)]/[(C_2_)^2^]), and between these hydrocarbons and n-butane, Q* (K = [(C_1_)*(n-C_4_)]/[(C_2_)*(C_3_)]), reported here for the first time. Two observations stand out. Initial hydrocarbon products are near equilibrium and have maximum average molecular weights (AMW). Over time, products fall from equilibrium and AMW in concert. It is consistent with metathesis splitting olefin intermediates [C_n_] to smaller intermediates (fission) as gas generation creates open catalytic sites ([ ]): [C_n_] + [ ] → [C_n-m_] + [C_m_]. Fission rates increasing exponentially with olefin molecular weight could contribute to these effects. AMW would fall over time, and selective fission of [C_3_] and [n-C_4_] would draw Q and Q* from equilibrium. The results support metathesis as the source of thermodynamic equilibrium in natural gas.

## Introduction

Thermal cracking has been accepted as the source of natural gas for decades [1,2]. Although alternatives have been proposed and deficiencies in thermal cracking theory cited [3], it has retained extraordinary allegiance over time. This was in spite of the fact that laboratory simulations had consistently failed to generate gas resembling natural gas [4-10]. Natural gas (C_1_-C_4_) contains about 80% wt methane while experimentally generated gas from thermal cracking was always depleted in methane, and remained so over prolonged periods of cracking [3]. Higher methane concentrations had been generated, but only at extraordinary temperatures (> 400°C) where ethane and propane decompose [11]. It has been argued that natural gas is generated depleted in methane, and becomes enriched in methane after generation by some unspecified fractionation [12,13]. But, there is no sign of the hypothetical heavy fraction in conventional reservoirs and no plausible explanation for its disappearance [3]. Thermal cracking has nevertheless been embraced as the primary source of natural gas and alternatives essentially dismissed as possible contributors [4-10].

This changed with the recent disclosure of gas generation at temperatures 300° below thermal cracking temperatures [14]. It was catalytic gas generated from marine shales under anoxic conditions, natural catalysis carried from the subsurface requiring no artificial stimulation. Shales generated gas in aperiodic episodes at ambient temperatures under inert gas flow. When gas was retained in closed reactors, it reached metathetic equilibrium in methane, ethane, and propane, and became enriched in methane over time [15]. Natural gas was also shown to be constrained to equilibrium in molecular and isotopic compositions. Other reports have shown counter-intuitive effects in low-temperature gas generation over time [16]. Shales released increasing concentrations of lighter hydrocarbons over time, the exact opposite to desorption or other simple first-order processes.

Here we address metathesis as a possible source of equilibrium in our experiments. The results and proposed mechanism are presented in reverse order, with the proposed kinetic scheme presented first as context for the experimental results that follow.

## Results and Discussion

### Proposed Kinetic Scheme

**Figure 1:**
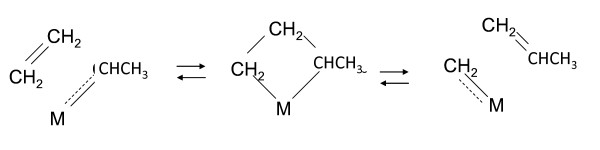
**Key intermediates in the proposed mechanism for low-temperature gas generation in marine shales**. M denotes a low-valent transitions metal capable of forming two metal-carbon bonds. The central complex is a metallocyclobutane interconverting with olefin-carbene-metal complexes, an ethylidene carbene complex to the left and a methylene carbene complex to the right. This is the Chauvin mechanism proposed for olefin metathesis [19].

All intermediates and reactions presented below are known in catalysis by low-valent transition metals [17]. Interconverting metallocyclobutanes, carbenes, and olefins are key intermediates in our scheme (Figure 1) as they are in olefin metathesis [18]. They are hydrogen deficient to one degree, either as olefins (ethylene to the left and propylene to the right), carbenes (ethylidene to the left and methylidene to the right), or metallocylobutanes (center). The reactions in Figure 1 were first proposed by Chauvin [19] to explain olefin metathesis, then referred to as 'olefin disproportionation', a remarkable catalytic reaction first discovered by Banks and Bailey in 1964 [20]. These intermediates have since gained recognition in a broad variety of hydrocarbon skeletal rearrangements including metathesis in various forms [17,18].

Intermediates are symbolized by [C_n_], where [ ] denotes an active open site (a metal) and C_n _an unsaturated hydrocarbon C_n_H_2n _bonded to it. [C_n_] in the following reactions does not infer a specific structure. It can be any one of the three structures in Figure 1, for example. Metathesis splits unsaturated hydrocarbons into two unsaturated hydrocarbons reversibly. For example, the metallocyclobutane in Figure 1, C_4_H_8 _is split into C_1_H_2 _+ C_3_H_6 _or 2 C_2_H_4_. It should not be confused with thermal cracking. These are unsaturated hydrocarbons bonded to transition metals. The interconversions are therefore low-energy interconversions that can proceed with facility at low temperatures.

Hydrocarbons enter a catalytic cycle by addition to an active open site, [ ], with a loss of hydrogen (rx. 1). The form of hydrogen is not specified except that it is not molecular hydrogen. It is hydrogen stored near active sites, labile hydrogen that can be delivered to the site to generate saturated hydrocarbons (rx. 2) or withdrawn from the site (rx. 1).(1)

C_n _in [C_n_] can metathesize (split) to smaller intermediates on a single metal site as Figure 1 illustrates. The olefin can also escape the site by transferring to an adjacent open site, a process referred to here as 'fission' (rx. 3):(3)

Thus, [C_4_] in Figure 1 can transfer either ethylene or propylene to an adjacent [ ], generating [C_2_] + [C_2_] or [C_1_] + [C_3_], respectively.

[C_n_] can also exchange olefin ligands with an adjacent intermediate bringing the partners to equilibrium (rx. 4) (the parentheses denote linkage, ↔ represents a reversible reaction):(4)

This is illustrated in rx. 5 representing the metathetic equilibrium between methane, ethane, and propane (K = [(C_1_)*(C_3_)]/[(C_2_)^2^]), and in rx. 6 for methane, ethane, propane, and n-butane (K* = [(C_1_)*(n-C_4_)]/[(C_2_)*(C_3_)]), both addressed experimentally below.(5)

Metathetic reactions occur between adjacent sites in this scheme. High concentrations of [ ] will promote fission through rx. 3 and high concentrations of [C_n_] will promote equilibrium through rx. 4. Both are metathesis reactions. Concentrations of [C_n_] and [ ] will control which of the two processes dominate and how they will effect product average molecular weight (AMW). Fission lowers AMW while equilibrium has little effect on it.

Under initial conditions, before gas generation, intermediates should be near equilibrium. The system would be saturated in [C_n_] and only reactions like 4 could proceed. Saturated hydrocarbons concentrated over catalyst surfaces (active surfaces) could interconvert with intermediates (reactions 1 & 2) bringing saturated hydrocarbons to equilibrium over time. The onset of gas generation with heating should therefore release hydrocarbons near equilibrium. It would also increase concentrations of [ ] promoting fission and products with lower MW over time.

Finally, there is the question of hydrogen source to sustain gas generation. Although it does not impact of the subject at hand, it is included here to complete the scheme. We view intermediates [C_n_] as the source of hydrogen through degradation (rx. 7).(7)

This hypothetical scheme best fits our experimental results and is used below as context for data presentation.

It is instructive at this point to consider a conventional model for the release of gas from carbonaceous shale under gas flow at low temperatures. We assume the shale is inert and gas is released by desorption. The source of gas is in-place saturated hydrocarbons: C_n _= C_n_H_2n+2_. [C_n_] denotes C_n_H_2n+2 _dissolved in kerogen and bitumen and adsorbed on inorganic surfaces. There are no pathways like rx. 3 to lower MW because thermal cracking rates at 100°C are essentially zero [21]. Heating will drive the lighter hydrocarbons from the shale before the heavier hydrocarbons. Thus, the gas desorbed from the shale will *increase *in MW over time, but the increase is a fractionation with no net change in MW. The *decrease *in MW in our scheme is due to fission with a net change in MW.

### Product Molecular Weights over Time

**Figure 2:**
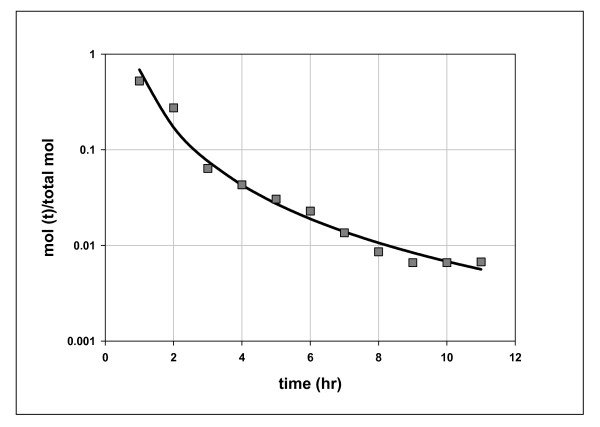
**Molar yields of C_1_-C_5 _hydrocarbons over time, Mowry shale, 11 hours at 100°C**. Data from Table 1 (Mowry 1) in mol fraction total product over time. The line through the data is the regression curve: y = 0.305 * t^-2.073^, where y = n mol product at time t/total n mol product; t = time. R^2 ^= 0.98.

**Figure 3:**
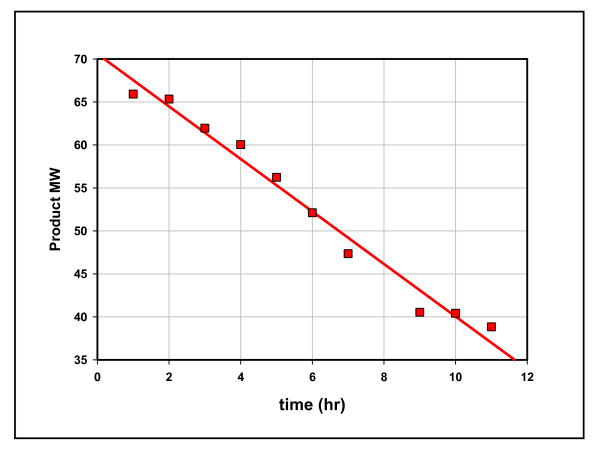
**Product molecular weight over time, Mowry shale, 11 hours at 100°C**. Data from Mowry 2, Table 1. Product MW is the total wt of hydrocarbons generated in one-hour segments divided by the total moles hydrocarbons (AMW) generated in that hour (wt/mol). The straight line is the linear regression line, R^2 ^= 0.98.

Cretaceous Mowry shale was heated isothermally (100°C) for 11 hours in closed reactors. Aliquots of product removed hourly are displayed in Table 1. Their compositions changed over time and the amounts of hydrocarbons in each aliquot fell sharply, as a power function of time (Figure 2). The continuous fall suggests a single episode of generation similar to the single episodes under gas flow, with yields rising and falling sharply but continuously over time [14]. The departure from exponential in Figure 2 suggests a complex catalytic process distinct from first-order thermal generation. Figure 3 shows the linear fall in product AMW over reaction time, consistent with preferential degradation of higher hydrocarbons.

**Table 1:** The distribution of C_1_-C_5 _hydrocarbons generated from Mowry shale, 100°C.

	**Mowry 1**										
**time (hr)**	**1**	**2**	**3**	**4**	**5**	**6**	**7**	**8**	**9**	**10**	**11**
**C_1_**	3.42	2.96	2.26	2.15	2.11	2.42	1.53	1.23	0.94	1.02	1.19
**C_2_**	0.87	0.83	0.53	0.52	0.58	0.68	0.42	0.34	0.39	0.34	0.32
**C_3_**	5.00	2.69	0.90	0.72	0.65	0.62	0.41	0.37	0.25	0.24	0.29
**i-C_4_**	25.74	12.65	3.16	1.84	1.09	0.61	0.31	0.20	0.10	0.10	0.10
**n-C_4_**	29.01	14.29	2.99	1.90	1.28	0.92	0.53	0.26	0.31	0.25	0.26
**CP**	12.55	6.87	1.34	0.91	0.64	0.37	0.24	0.17	0.08	0.08	0.08
**i-C_5_**	72.07	37.75	8.26	5.18	3.26	1.88	1.01	0.38	0.25	0.25	0.08
**n-C_5_**	54.42	28.27	5.31	3.44	2.23	1.38	0.81	0.37	0.24	0.28	0.27
**Sum mol**	203.08	106.31	24.76	16.67	11.83	8.87	5.26	3.32	2.56	2.56	2.61
**Sum wt**	13462	6987	1520	984	657	435	252	139	102	101	94
**Product AMW**	66	66	61	59	56	49	48	42	40	40	36
**log Q**	1.35	1.07	0.86	0.75	0.61	0.50	0.55	0.60	0.18	0.31	0.52
log Q*	1.36	1.28	1.15	1.04	0.85	0.72	0.66	0.41	0.47	0.49	0.51
											
	**Mowry 2**										
**time (hr)**	**1**	**2**	**3**	**4**	**5**	**6**	**7**	**8**	**9**	**10**	**11**
**C_1_**	2.78	2.70	2.08	1.72	1.73	1.61	1.69		0.98	1.15	0.88
**C_2_**	0.81	0.92	0.64	0.44	0.52	0.48	0.52		0.28	0.35	0.28
**C_3_**	9.31	4.34	1.19	0.79	0.68	0.54	0.53		0.25	0.29	0.24
**i-C_4_**	31.63	13.39	2.71	1.46	0.81	0.47	0.32		0.11	0.12	0.06
**n-C_4_**	43.35	18.12	3.49	1.94	1.29	0.88	0.71		0.29	0.34	0.27
**CP**	14.59	7.31	1.70	1.01	0.67	0.40	0.26		0.10	0.10	0.05
**i-C_5_**	80.07	37.15	8.27	4.61	2.77	1.58	1.01		0.24	0.26	0.18
**n-C_5_**	68.80	31.18	6.49	3.63	2.23	1.33	0.89		0.28	0.36	0.23
**Sum mol**	251.35	115.11	26.57	15.60	10.70	7.30	5.93		2.52	2.97	2.17
**Sum wt**	16568	7521	1646	936	602	381	281		102	120	84
**Product AMW**	66	65	62	60	56	52	47		41	40	39
**log Q**	1.59	1.14	0.78	0.84	0.64	0.58	0.52		0.49	0.45	0.43
log Q*	1.20	1.09	0.98	0.98	0.80	0.74	0.64		0.61	0.59	0.55

Data in mol × 10^9 ^hydrocarbon/g shale in a 2 ml aliquot collected at the hour indicated. Sum wt is wt C_1_-C_5 _hydrocarbons generated in the hour indicated, (g/g shale hr) × 10^9^. Product AMW is the wt product divided by moles product: Sum wt/Sum mol. Log Q is log ([(C_1_)*(C_3_)]/[(C_2_)^2^]). Log Q* is log ([(C_1_)*(n-C_4_)]/[(C_2_)*(C_3_)]).

**Figure 4:**
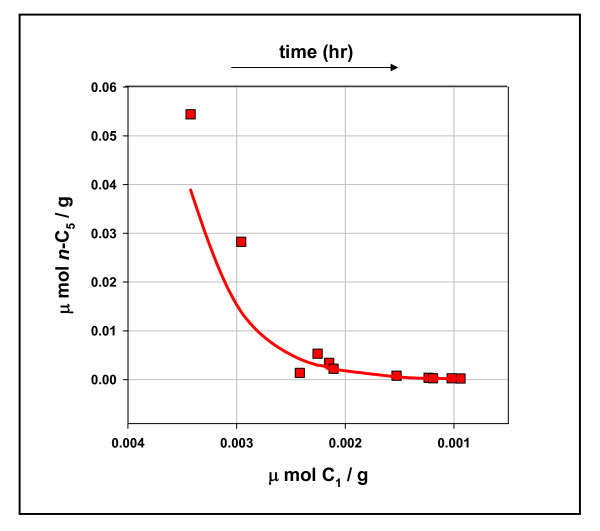
**The exponential fall in n-pentane yield relative to methane yield, Mowry shale, 11 hours at 100°C**. Concentrations (μmol/g shale hr) are yields over time (Table 1). The red curve is the regression line for y = (2 × 10^-5^)* e^2212*x^, where y = n-C_5 _concentrations and x = C_1 _concentrations; R^2 ^= 0.93. Ethane through n-pentane show the same exponential relationship to methane with decreasing exponents α: n-C_5_, α = 2212; n-C_4_, α = 1924, R^2 ^= 0.91; C_3_, α = 1139, R^2 ^= 0.93; C_2_, α = 416, R^2 ^= 0.93.

**Figure 5:**
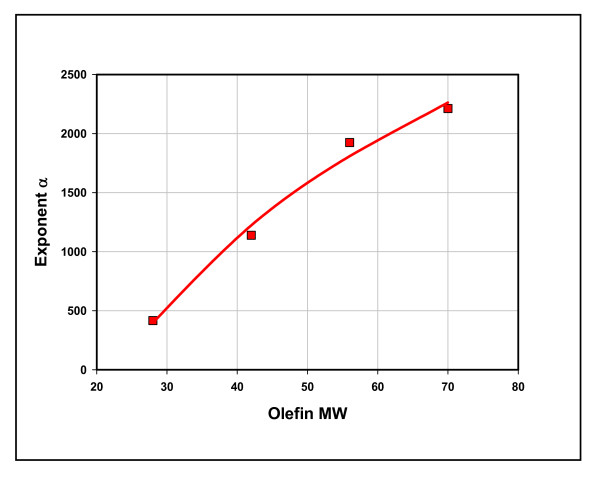
**The relationship between exponential decline (Figure. 4) and molecular weight of olefin intermediates, Mowry shale, 11 hours at 100°C**. Alpha along the y axis is the exponent to the exponential curves given in the legend of Figure. 4. Olefin MW represents the molecular weights of C_2 _to C_5 _olefins, the intermediates in [C_n_]. The red line is the regression curve: y = 2035*ln(x) - 6385; R^2 ^= 0.99.

Methane is a terminal product in our mechanism. With the exception of degradation to carbon and hydrogen (rx. 7), [C_1_] can only increase in concentration relative to higher hydrocarbons. Figure 4 is a plot of methane vs n-pentane (μmol/g shale hr) with the time direction indicated above the figure. Both rates fall with time, but the higher hydrocarbon falls exponentially. All hydrocarbons show this relationship with methane. There is one significant difference, however. The exponent α in C_n _= Ae^αC1 ^shows a strong logarithmic correlation to C_n _MW shown in Figure 5 (R^2 ^= 0.99). We attribute this to rates of fission *increasing *with intermediate MW. Olefin bonds to transition metals weaken with molecular weight [18]. Rates of olefin transfer, therefore, should *increase *with MW. Thus, rates of fission should increase with MW. We explain the strong correlation in Figure 4 to diminishing coordinate bond strength with increasing olefin MW.

Other factors could contribute as well including the preferential adsorption of higher hydrocarbons to active surfaces. The possibility of thermal anomalies must also be considered. Since generation is probably exothermic [14], larger amounts of heat released early in the reaction could dislodge disproportionate amounts of saturated higher hydrocarbons from active surfaces. Early episodes of thermal desorption would inflate early hydrocarbon yields thus contributing to an artificial decline in yields over the first hours of reaction.

### Metathetic Equilibria over Time

**Figure 6:**
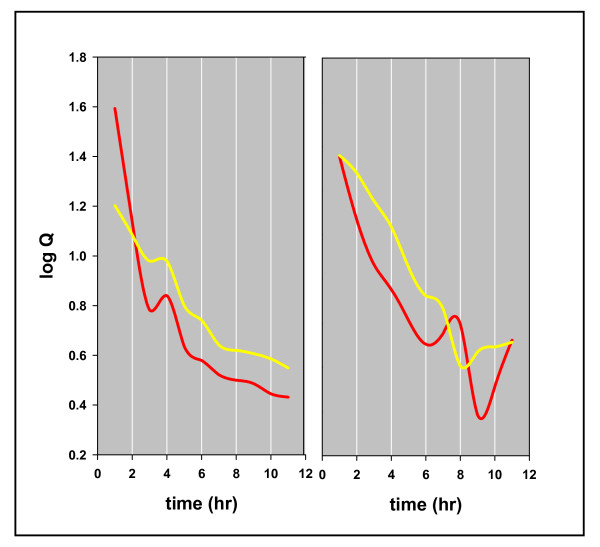
**The fall in metathetic equilibria over time in duplicate experiments, Mowry shale, 11 hours at 100°C**. The left panel represents Mowry 1 and the right Mowry 2, Table 1. The red lines trace Q = [(C1)*(C3)/(C2)2] and the yellow lines trace Q = [(C1)*(n-C4)/(C2)*(C3)].

We addressed two metathetic equilibria (rx. 4) in these experiments, that between methane, ethane, and propane (K = [(C_1_)*(C_3_)]/[(C_2_)^2^]) and between methane, ethane, propane, and n-butane (K* = [(C_1_)*(n-C_4_)]/[(C_2_)*(C_3_)]). Thermodynamic equilibrium at 100°C is log K = 1.17 for methane, ethane, and propane, and log K = 1.62 for methane, ethane, propane, and n-butane [22]. Figure 6 traces both quotients over time in duplicate experiments. Near thermodynamic equilibrium in the first hour (log Q = 1.5 and 1.3, respectively), both quotients fall sharply from equilibrium over time.

**Figure 7:**
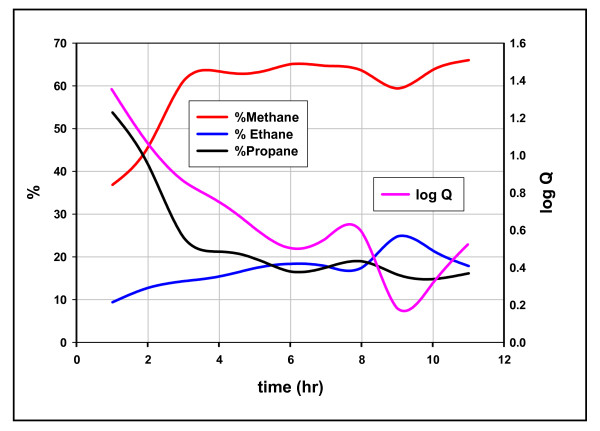
**The composition of methane, ethane, and propane over time and its relation to equilibrium, Mowry shale, 11 hours at 100°C**. Q = [(C_1_)*(C_3_)/(C_2_)^2^], % vol in C_1_-C_3_. The log Q curve (the right y axis) is superimposed on the three % vol curves (left y axis).

**Figure 8:**
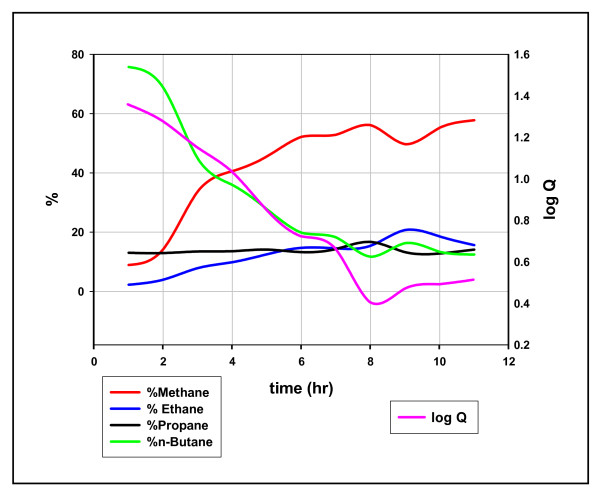
**The composition of methane, ethane, propane, and n-butane over time and its relation to equilibrium, Mowry shale, 11 hours at 100°C**. Q = [(C_1_)*(*n*-C_4_)/(C_2_)*(C_3_)], % vol in C_1_-C_4_. The log Q curve (the right y axis) is superimposed on the four % vol curves (left y axis).

**Figure 9:**
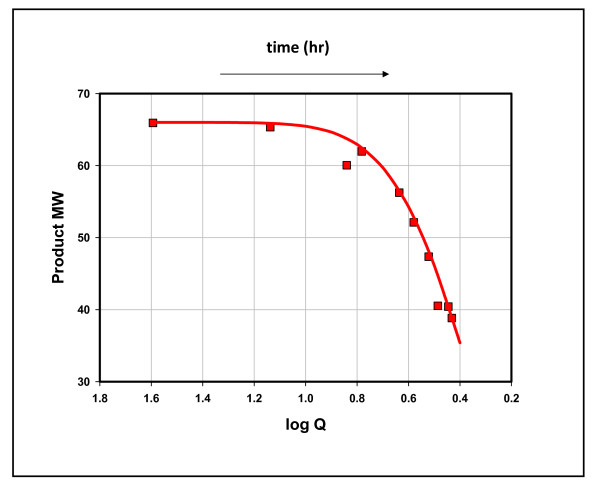
**Product average molecular weight and equilibrium (log Q), Mowry shale, 11 hours at 100°C**. Data (Product AMW) from Mowry 2, Table 1. The line through the curve was constructed to fit the data: y = 66 * (1-e^-4.8*(*logQ*)2^).

Each plot shows coherent oscillations linking the two quotients, a characteristic of these reactions noted before [14]. Figures 7 &8 show the concentrations of hydrocarbons superimposed on the quotients. In each example, the fall from equilibrium can be attributed to sharp declines in the heavier hydrocarbon, propane in Figure 7 and n-butane in Figure 8. These results are in accordance with the above scheme: open site concentrations increase with generation promoting fission and diminishing *adjacent *occupied site concentrations suppress a restoration to equilibrium. Product AMW and Q are linked to [ ] in the scheme and therefore should be linked to each other. Figure 9 shows that they are.

Extraordinary correlations attend gas generation from the Mowry shale over time. Table 2 lists the coefficients of correlation (R^2^) between five mathematically independent variables. The linear correlation between log Q and log Q* (0.81) is consistent with fission (rx. 3) driving both quotients from equilibrium in concert. Fission also explains the correlation between AMW and log Q (Figure 9) and the relationship between olefin MW and rates of generation as reflected in Figure 5.

**Table 2:** Coefficients of correlation (R^2^) between five reaction variables, Mowry shale, 11 hours at 100°C.

		log Q	log Q*	Product AMW	Sum wt	time
**log Q**		1				
log Q*		0.81	1			
**Product AMW**		0.7	0.95	1		
**Sum wt**		0.88	0.95	0.89	1	
**time**		-0.67	-0.9	-0.98	-0.98	1

Data was taken from Mowry 1, Table 1. Most correlations (R^2^) are nonlinear. The coefficients of correlation are for curves that best fit the data: log Q vs Sum wt fits an exponential curve; log Q vs time fits a power curve; Sum wt vs time fits an exponential curve; Product AMW vs time fits a logarithmic curve.

## Conclusions

Gas generated from Mowry shale at 100°C is catalytic gas generated through metathesis. A number of correlations (Table 2) support this view. Product AMW, equilibrium quotients, yields, and reaction times all correlate. A kinetic scheme is proposed in which gas generation promotes lower MW products over time with a fall from equilibrium. The experimental results fit this scheme remarkably well. Metathesis catalyzed by low-valent transition metals is very likely the source of gas in these experiments. It provides an attractive alternative to thermal cracking as the source of natural gas. It is in many respects superior. Metathesis explains three properties of natural gas that are not easily explained otherwise: thermodynamic equilibrium [15], high methane concentrations [3], and evolution of wet gas to dry gas over geologic time [16].

## Experimental

The Cretaceous Mowry shale is whole core (2500 m) from an unknown well in Colorado. Rock-Eval: S1 = 2.61 mg hydrocarbon/g rock; S2 = 9.33 mg hydrocarbon/g rock; S3 = 0.15 mg CO_2_/g rock; Tmax = 439°C. Total Organic Carbon (Leco) = 2.5%. The experimental procedures for sample preparation and product analysis are described elsewhere [14]. Closed experiments were carried out in 5 ml glass vials fitted with PTFE/SIL septa purchased from Cobert Ass. Samples ground to 60 mesh under argon (~1 g) were placed in vials under argon, and sealed with open screw caps fitted with septa. Caps were secured to the vials with plastic electrical tape to prevent leakage under heating. Vials were heated in a convection oven at 100°C (± 5°) for 11 hours under argon. Aliquots of product gas were removed hourly by inserting two needles into the vial through the septum; one withdrew 2 ml of gas into a gas-tight syringe and the other injected 2 ml argon into the vial to replace the gas withdrawn. Gas was withdrawn and injected simultaneously (the injecting needle was under moderate argon pressure) with the injecting needle near the septum and the withdrawing needle near the shale. The gas samples were analyzed and discarded. The results of duplicate experiments are in Table 1. Duplicate experiments did not use aliquots of 60 mesh shale. Different samples from the same source were subjected to the same experimental procedures: grinding in argon, sieving, and so forth. The variations in yield and product compositions shown in Table 1 therefore reflect heterogeneity in sample, variance in sample preparation, and product analysis.

## Competing interests

The authors declare that they have no competing interests.

## Authors' contributions

FM formulated theory, and both authors contributed to the experimental work and the final paper.
